# GRP78 Suppresses Lipid Peroxidation and Promotes Cellular Antioxidant Levels in Glial Cells following Hydrogen Peroxide Exposure

**DOI:** 10.1371/journal.pone.0086951

**Published:** 2014-01-24

**Authors:** Kaori Suyama, Masahiko Watanabe, Kou Sakabe, Asako Otomo, Yoshinori Okada, Hayato Terayama, Takeshi Imai, Joji Mochida

**Affiliations:** 1 Department of Anatomy and Cellular Biology, Basic Medical Science, Tokai University School of Medicine, Isehara, Kanagawa, Japan; 2 Department of Orthopedic Surgery, Surgical Science, Tokai University School of Medicine, Isehara, Kanagawa, Japan; 3 Department of Molecular Life Science, Basic Medical Science, Tokai University School of Medicine, Isehara, Kanagawa, Japan; 4 Tokai University Teaching and Research Support Center, Isehara, Kanagawa, Japan; Massachusetts General Hospital/Harvard Medical School, United States of America

## Abstract

Oxidative stress, caused by the over production of reactive oxygen species (ROS), has been shown to contribute to cell damage associated with neurotrauma and neurodegenerative diseases. ROS mediates cell damage either through direct oxidation of lipids, proteins and DNA or by acting as signaling molecules to trigger cellular apoptotic pathways. The 78 kDa glucose-regulated protein (GRP78) is an ER chaperone that has been suggested to protect cells against ROS-induced damage. However, the protective mechanism of GRP78 remains unclear. In this study, we used C6 glioma cells transiently overexpressing GRP78 to investigate the protective effect of GRP78 against oxidative stress (hydrogen peroxide)-induced injury. Our results showed that the overexpression of GRP78 significantly protected cells from ROS-induced cell damage when compared to non-GRP78 overexpressing cells, which was most likely due to GRP78-overexpressing cells having higher levels of glutathione (GSH) and NAD(P)H:quinone oxidoreductase 1 (NQO1), two antioxidants that protect cells against oxidative stress. Although hydrogen peroxide treatment increased lipid peroxidation in non-GRP78 overexpressing cells, this increase was significantly reduced in GRP78-overexpressing cells. Overall, these results indicate that GRP78 plays an important role in protecting glial cells against oxidative stress via regulating the expression of GSH and NQO1.

## Introduction

Reactive oxygen species (ROS) are one of the cytotoxic factors produced from damaged cells that cause oxidative stress and tissue damage during neurotrauma [1]. Hydrogen peroxide (H_2_O_2_), a ROS, is released from dying cells during neurotrauma and neurodegenerative disease and causes tissue destruction [2], [3]. H_2_O_2_ can produce hydroxyl radicals (OH·) and mediate cell damage either through direct oxidation of lipids, proteins and DNA or act as a signaling molecule to trigger cellular apoptotic pathways [4]–[6]. Therefore, it is important to protect cells from H_2_O_2_-induced cell damage as a therapeutic strategy against neurotrauma and neurodegenerative diseases [7], [8]. Endoplasmic reticulum (ER) stress has been reported to be one of the pathways via which cells are damaged and die following ROS exposure [9], [10].

The mechanism by which ER stress promotes apoptosis in cells hinges on driving the accumulation of structurally abnormal proteins that are usually repaired by ER chaperones to prevent cell death [11]. The 78 kDa glucose-regulated protein (GRP78) is one example of an ER chaperone that regulates protein folding in the ER and controls the ER-Ca^2+^ balance via trans membrane ER stress sensors, which contribute to cell survival [11]–[13]. GRP78 has been suggested to not only protect cells against high-concentrations of glutamate or tunicamycin, which induce ER stress directly [14], but also protect cells from ROS damage [15]–[17].

Many studies have focused on various antioxidant factors, such as glutathione and NAD(P)H:quinone oxidoreductase 1 (NQO1). A previous study reported that induction of NQO1 and GSH by dimethyl fumarate, 3H-1,2-dithiole-3-thione or tert-butylhydroquinone (tBHQ) protected against neurocytotoxicity caused by dopamine, 6-hydroxydopamine, 4-hydroxy-2-nonenal,or H_2_O_2_ [18]. As this study described, these antioxidants have recently been demonstrated to play an important role in protecting cells against oxidative stress [19]–[21]. Glutathione is the most abundant low molecular weight thiol in most organisms [20], [22]. There are two types of glutathione, reduced glutathione (GSH) and oxidized glutathione (GSSG), depending on the environment. Reduced glutathione (GSH) is the main non-protein antioxidant and plays a critical role in the detoxification of H_2_O_2_ and lipid hydroperoxide, and is involved in the protection against oxidative stress [18]. Similarly, NQO1, one of the most extensively investigated phase 2 enzymes, is an effective antioxidant that protects membrane phospholipids from oxidative damage and plays an important protective role in oxidative stress [19].

Few studies have investigated the influence of GRP78 on NQO1. Some studies have suggested that H_2_O_2_ may not be involved in ER stress-dependent cell damage because the response of GRP78 is different following H_2_O_2_ exposure and other cytotoxic factors [23], [24]. Similarly, a report on PKE-like ER kinase (PERK), a ER-stress sensing protein that resides in the ER, suggested that the PERK pathway is activated after dissociation of GRP78 from PERK monomers and leads to intracellular GSH production [25]. As these studies showed, the role of GRP78 during oxidative stress remains unclear. Therefore, we used cells transiently overexpressing GRP78 to investigate the protective effect of GRP78 against high extracellular concentrations of H_2_O_2_ and evaluated the response of glutathione and NQO1.

## Materials and Methods

### Cell Culture and Culture Conditions

The rat C6 glioma cell line was obtained from American Type Culture Collections (ATCC, Manassas, VA, USA). Cells were plated onto 6 cm tissue culture dishes and maintained at 37°C with 5% (v/v) CO_2_ and 95% (v/v) air. The culture medium consisted of Dulbecco’s modified Eagle’s medium (DMEM; Gibco/Life Technologies, Carlsbad, CA, USA), 10% (v/v) fetal bovine serum (FBS), and 1% (v/v) penicillin–streptomycin.

### H_2_O_2_ Treatment of C6 Cells

C6 cells were plated onto 6 cm tissue culture dishes at a density of 2.0×10^6^ cells/dish, incubated for 36 h and then treated with fresh medium containing 1–6 mM H_2_O_2_ (Wako, Osaka, Japan) for 6 and 24 h.

### Antibodies

Antibodies used were: rabbit polyclonal antibody against GRP78 (AnaSpec Inc. Fremont, CA USA); goat polyclonal antibody against GRP78 (Santa Cruz Biotechnology, Inc. Dallas, TX, USA); rabbit polyclonal antibody against NAD(P)H:quinone oxidoreducrase1(NQO1) (Santa Cruz Biotechnology Inc.); mouse monoclonal antibody against NAD(P)H:quinone oxidoreducrase1(NQO1) (Novus Biologicals, LLC. Littleton, CO. USA); mouse monoclonal antibody against β-actin (Sigma-Aldrich Inc. St. Louis, MO, USA); Alexa 488-conjugated anti-rabbit antibody; Alexa 488-conjugated anti-mouse antibody (Invitrogen/Life Technologies); PE-conjugated anti-mouse secondary antibody (BioLegend Inc. San Diego, CA, USA); horseradish peroxidase (HRP)-conjugated goat polyclonal antibody against rabbit IgG; HRP-conjugated goat polyclonal antibody against mouse IgG; HRP-conjugated rabbit polyclonal antibody against goat IgG (DAKO Glostrup, Denmark).

### Intracellular GRP78 and NQO1 Detection

The IntraStain^®^ reagent kit (DAKO) has previously been used successfully for the detection of intracellular GRP78 or NQO1 protein expression under conditions of tunicamycin treatment and βactin [14]. Following exposure to treatment, cells were fixed and permeabilized using IntraStain according to the manufacturer’s instructions. For the detection of cytoplasmic GRP78 or NQO1, cells were stained with rabbit polyclonal anti-GRP78 or mouse monoclonal anti-NQO1, and then stained with the Alexa 488-conjugated anti-rabbit secondary antibody or PE-conjugated anti- mouse secondary antibody. GRP78 expression and β-actin expression were measured with a BD LSRFortessa™ apparatus (Becton Dickinson, Franklin Lakes, NJ, USA) and analyzed using FlowJo ™ Software (Tree Star Inc. Ashland, OR, USA).

### Construction of Chimeric Proteins and Transfection

**Figure 1:**
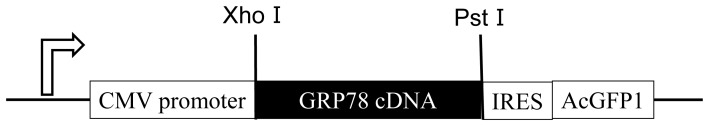
Schematic illustration of the pIRES2-AcGFP1 plasmid constructs used in the present study. cDNA of the rat GRP78 carried the XhoI/PstI cut sites and was cloned into the pIRES2-AcGFP1 plasmid.

The pIRES2-AcGFP1 plasmid was purchased from Clontech Laboratories. The plasmid contains the internal ribosome entry site (IRES) of the encephalomycarditits virus between the multiple cloning site (MCS) and the Aequorea coerulescens green florescent protein (Ac GFP) cording region. This permits both the gene of interest and the Ac GFP1 gene to be translated to a single bicistronic mRNA. A 1.9-kb cDNA of rat GRP78 (GenBank M14059), covering a whole open reading frame, was composed by Takara Bio. Inc. (Otsu, Japan) carrying XhoI/PstI cut sites and coned into pIRES2-AcGFP1 plasmid (Fig. 1). The pIRES-AcGFP1 vector was designed so that cells transfected transiently express GFP and the protein. Therefore, cells transfected with the GRP78 gene and expressing the protein express GFP. C6 cells were transfected with the indicated plasmids using the Neon™ transfection system (Invitrogen/Life Technologies). After transfection, cells consisting of GFP-positive and -negative cells were plated onto 6 cm tissue culture dishes at a density of 2.0×10^6^ cells/dish and incubated in DMEM with 10% (v/v) FBS for 36 h. Cells were then treated with fresh medium containing 1–6 mM of H_2_O_2_ for 6 h.

### Western Blot Analysis

After 36 h of transfection, GFP-positive and -negative cells were sorted using the FACSAria™ apparatus (Becton Dickinson, San Jose, CA, USA). Total extracted proteins from cultured cells were quantified using the Lowry method. Equal amounts of total protein from each sample were loaded onto a 12.5% (w/v) SDS–polyacrylamide gel and transferred to polyvinylidene difluoride (PVDF) membranes (Millipore, Billerica, MA, USA). Membranes were then incubated with the primary antibodies at 4°C overnight, followed by incubation with the HRP-linked anti-rabbit IgG (DAKO), anti-goat IgG (DAKO), or anti-mouse IgG (DAKO) antibodies for 1 h. Signals were detected using Immobilon Western Chemiluminescent HRP (Millipore). Intensity of specifically amplified products was quantified by densitometric scans of the films using computer software for a Macintosh “CS analyzer” (ATTO, Tokyo, Japan).

### Annexin V/Propidium Iodide (PI) Double Staining

Cells undergoing apoptosis or necrosis can be stained and quantified using Annexin V and propidium iodide (PI). Cells treated with H_2_O_2_, were washed once with phosphate buffered saline (PBS), and stained for 15 min with allophycocyanin-conjugated (APC)–Annexin V and PI (Becton Dickinson), according to the manufacturer’s instructions. Quantification of apoptotic/necrotic cell-death staining with APC–annexin V and PI under each condition were measured with a BD LSRFortessa™ apparatus (Becton Dickinson) and analyzed using FlowJo ™ Software (Tree Star Inc. Ashland, OR, USA). Annexin V-negative/PI-negative cells were considered living cells.

### Evaluation of Lipid Peroxidation

The lipid peroxidation of the cell membrane after H_2_O_2_ exposure was examined using cis-parinaric acid as a probe. Oxidation of this probe is accompanied by decreased fluorescence and absorption [26], [27]. cis-Parinaric Acid® was purchased from Molecular Probes/Life Technologies and the method was performed with slight modification as described by Hedley et al. [26]. Briefly, at 5 h after incubation with H_2_O_2_, 10 µM of cis-Parinaric Acid was added and the incubation was continued for 1 h (total incubation time was 6 h). Cells were collected with PBS and the fluorescence was measured using the BD LSRFortessa™ apparatus (Becton Dickinson) and analyzed using FlowJo ™ Software (Tree Star Inc.) The excitation and emission wavelengths were 320 and 420 nm, respectively.

### GSH Assay

Cellular GSH levels were monitored and analyzed using the ThiolTracker Violet GSH detection reagent® (Molecular Probes/Life Technologies) according to the manufacturer’s protocol. Cells were seeded in 6 cm dishes, and treated with 1–6 mM H_2_O_2_ for 6 h. After designated treatment times, cells were washed with Dulbecco’s PBS and incubated in Dulbecco’s PBS containing 10 µM (final concentration) ThiolTracker Violet for 30 min at 37°C. Cells were measured using the BD LSRFortessa™ apparatus (Becton Dickinson) and analyzed using FlowJo ™ Software (Tree Star Inc.). The excitation and emission wavelengths were 404 and 526 nm, respectively.

### Statistical Analysis

Data are expressed as the mean ± standard deviation (SD). Analysis of variance and the Student’s *t*-test were used to assess differences between the means of test samples and controls. Significance was set at *P*<0.05.

## Results

### Cell Damage and GRP78 Expression Following H_2_O_2_ Treatment

**Figure 2:**
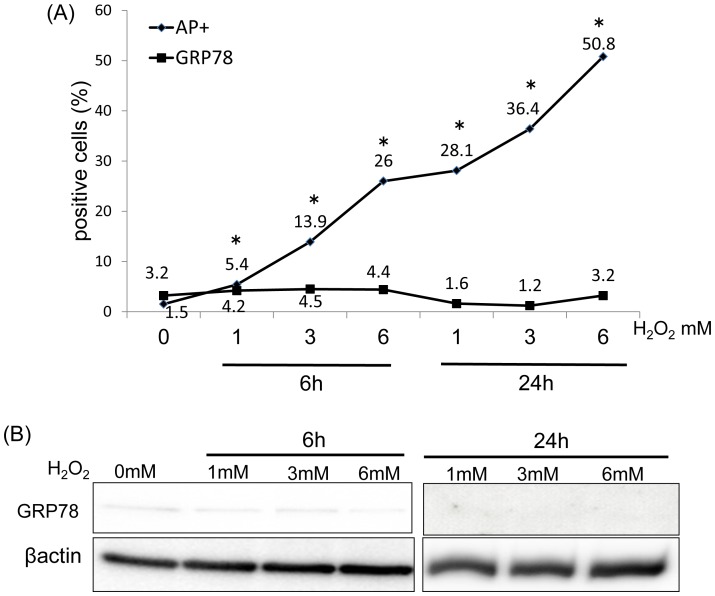
GRP78 expression in C6 cells following treatment with H_2_O_2_. (A) FACS analysis revealed a significant increase in the number of AP-positive cells following treatment with H_2_O_2_ when compared with untreated C6 cells (*P<0.05). Compared with untreated C6 cells, no significant differences were observed in GRP78 protein levels at any H_2_O_2_ concentration. (B) Western blot analysis of GRP78 in C6 cells treated with H_2_O_2_ for 6 h and 24h. There were no obvious changes in GRP78 expression levels.

Flow cytometric (FACS) analysis revealed a significant increase in the number of apoptotic/necrotic cells, namely annexin V/PI-positive cells (AP-positive cells) following 1–6 mM H_2_O_2_ treatment at 6∼24 h compared to that in the controls (Fig. 2A). The number of GRP78-expressing cells following treatment with H_2_O_2_ did not significantly increase compared to that in untreated C6 cells at any concentration (Fig. 2A). Similarly, western blot analysis revealed that the expression of the GRP78 protein in the groups treated with 1–6 mM H_2_O_2_ for 6 and 24 h did not increase obviously when compared to untreated C6 cells (n = 3; Fig. 2B).

### Evaluation of cis-Parinaric Acid and GSH Following H_2_O_2_ Treatment

**Figure 3:**
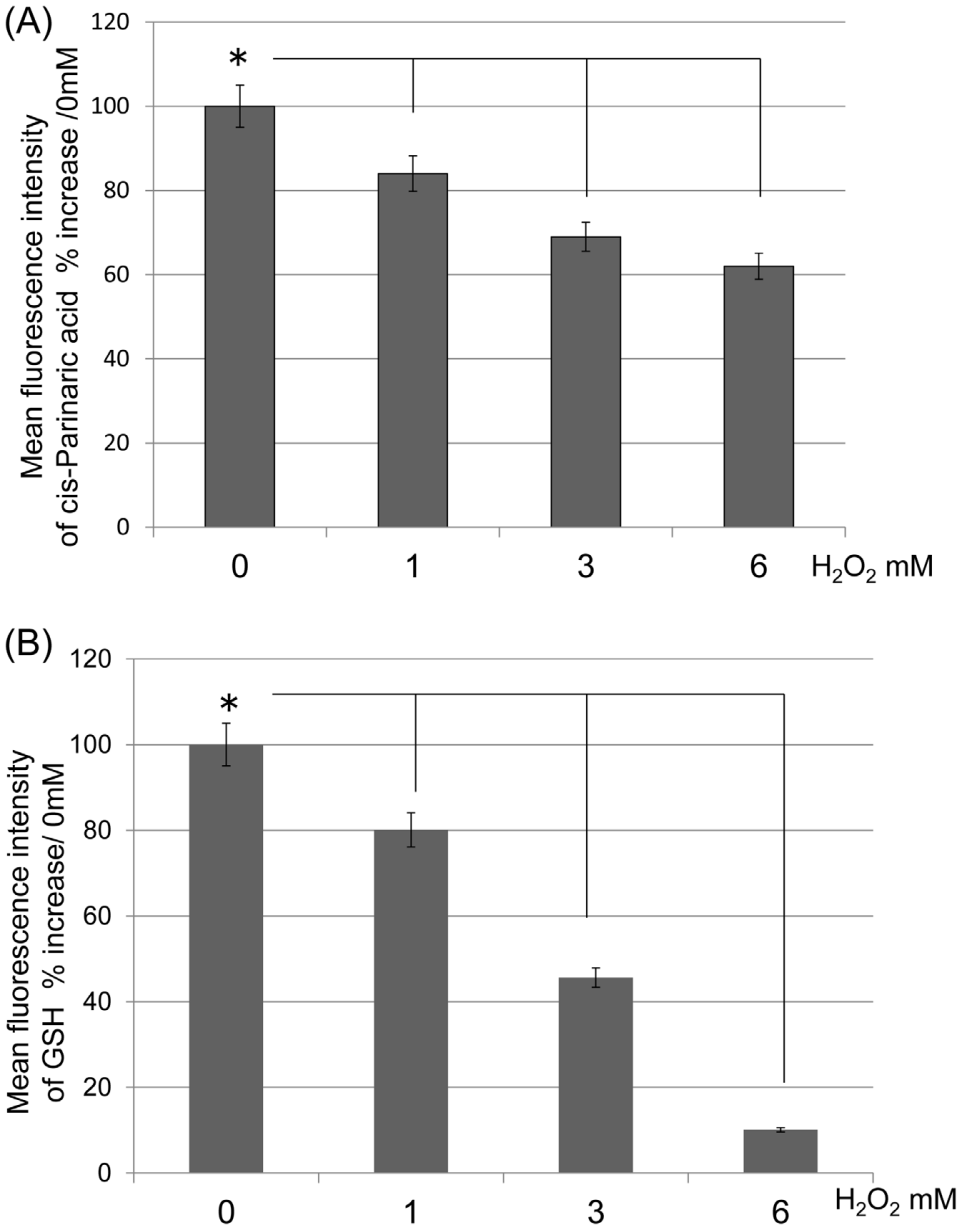
The mean fluorescence intensity of C6 cells. (A) The ratio of the mean fluorescence intensity of cis-Parinaric acid increased following treatment with H_2_O_2_ (*P<0.05). The value of 0 mM was designated as 100%. (B) The ratio of mean fluorescence intensity of reduced glutathione (GSH) following treatment with H_2_O_2_ (*P<0.05). The value of 0 mM was designated as 100%.

FACS analysis revealed that the mean fluorescence intensity of cis-Parinaric acid significantly decreased following treatment at all concentrations of H_2_O_2_ when compared to untreated C6 cells (n = 5; P<0.05) (Fig. 3A). This result indicated that H_2_O_2_ causes lipid peroxidation of the cell membrane. Similarly, the mean fluorescence intensity of GSH significantly decreased following treatment at all concentrations of H_2_O_2_ when compared with untreated C6 cells (n = 4; P<0.05) (Fig. 3B). These results indicated that intracellular GSH expression levels reduced when cells were subjected to excessive oxidative stress.

### Expression of GRP78 Protein and NQO1 in GFP-positive Cells after Transfection

**Figure 4:**
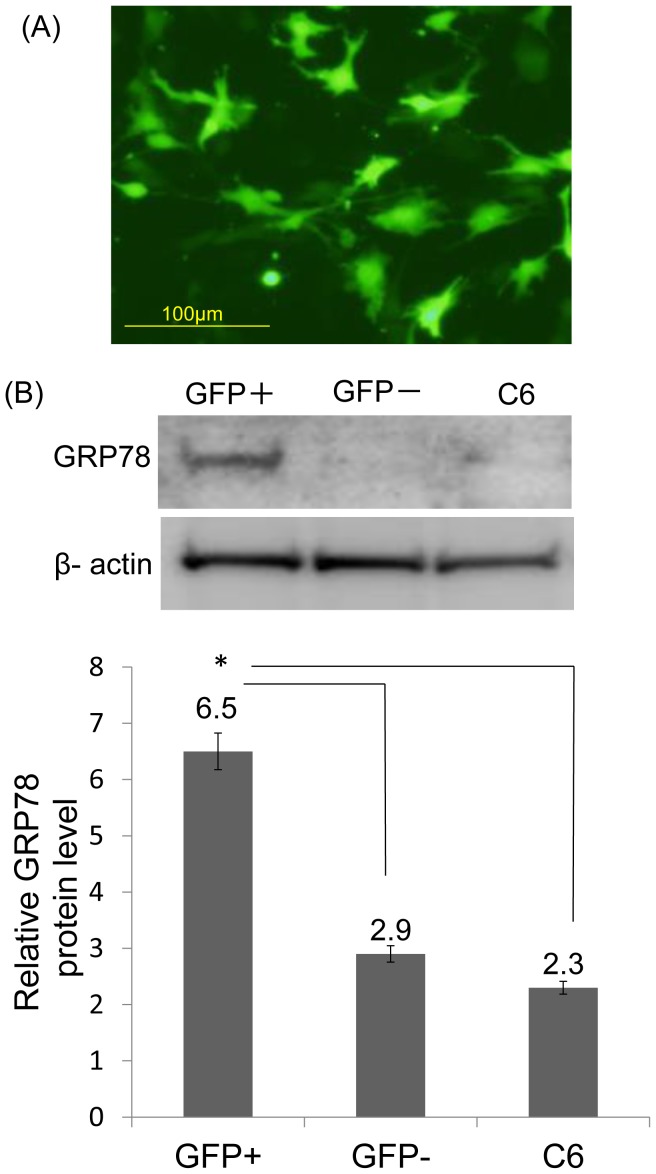
Expression of GRP78 protein in GFP-positive cells after transfection. (A) After 36 h of transfection, an average of 43% of cells was GFP-positive. (B) Quantification of western blots revealed that the GFP-positive cells expressed GRP78 protein prior to treatment with H_2_O_2_ compared with GFP-negative cells and untreated C6 cells (*P<0.05).

**Figure 5:**
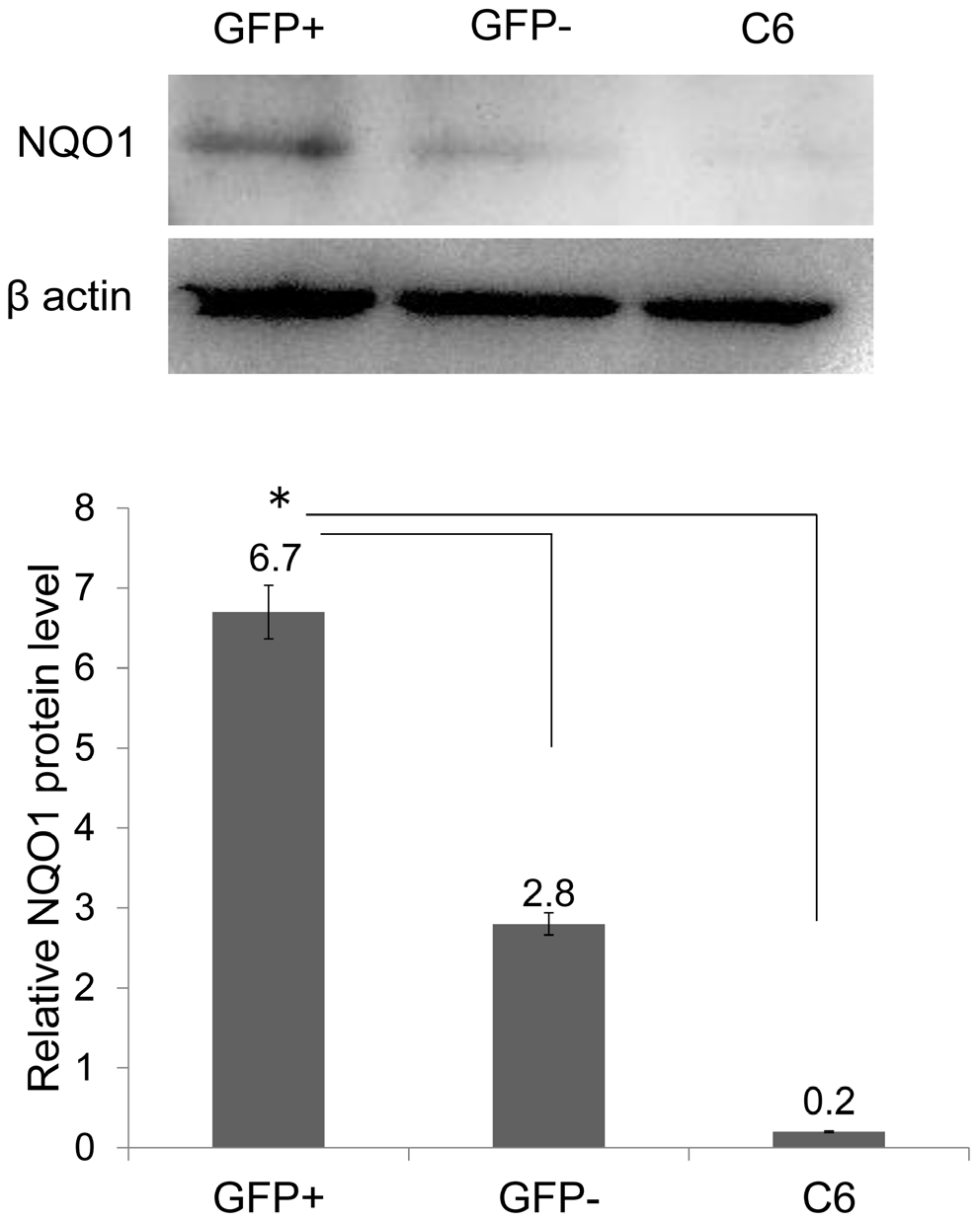
Expression of NQO1 in GFP-positive cells after transfection. Quantification of western blots revealed that GFP-positive cells expressed NQO1 protein prior to treatment with H_2_O_2_ when compared with GFP-negative cells and untreated C6 cells (*P<0.05).

At 36 h post-transfection, an average of 43% of cells was GFP-positive (Fig. 4A). Quantitation of immunoblots of GFP-positive cell extracts revealed a significant increase in GRP78 protein levels when compared with GFP-negative cells and non-treated C6 cells (n = 3, P<0.05) (Fig. 4B). This result showed that GFP-positive cells overexpress GRP78 protein before exposure to H_2_O_2_ when compared with GFP-negative cells and non-treated C6 cells. Moreover, we observed that NQO1 expression significantly increased in GFP-positive cell extracts when compared with GFP-negative cells and non-treated C6 cells (n = 3, P<0.05) (Fig. 5).

### Evaluation of GRP78 Overexpressing Cells Following H_2_O Exposure

**Figure 6:**
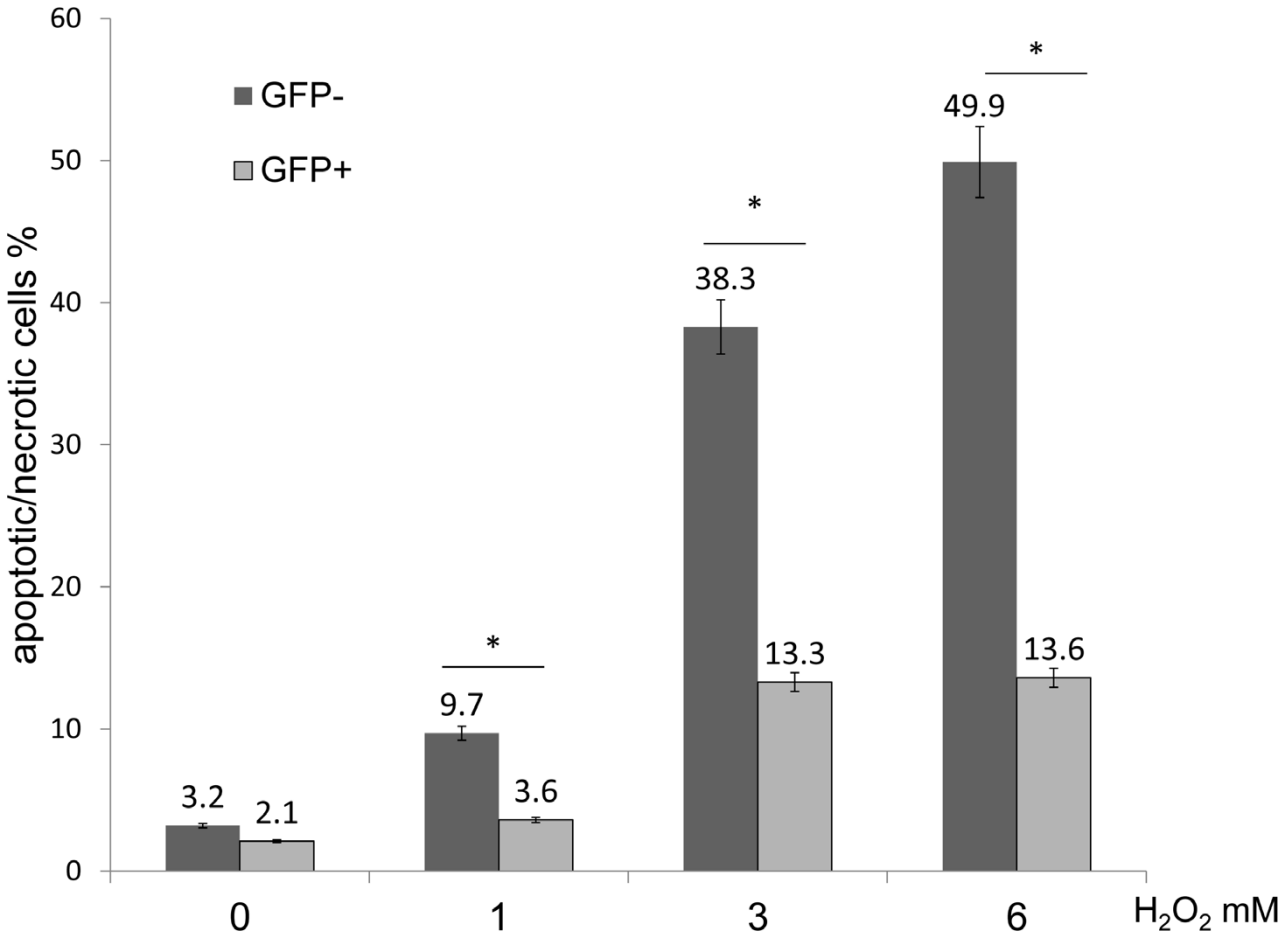
Evaluation of GRP78 overexpressing cells following H_2_O exposure. FACS analysis of GFP-negative and -positive cells labeling positive for Annexin V/PI following treatment with H_2_O_2_. The percentage of GFP-positive cells that were AP-positive significantly decreased when compared with GFP-negative cells following treatment with 1, 3 and 6 mM H_2_O_2_ (*P<0.05).

After transfection, a mixture of cells consisting of GFP-positive and -negative cells were incubated for 36 h with 10% (v/v) FBS/DMEM. Cells were then treated with 1–6 mM H_2_O_2_ for 6 h. To evaluate the protective effect of GRP78, apoptotic/necrotic cells were labeled with annexin V and PI, and the ratio of AP-positive cells in GFP-positive and -negative cells were analyzed byFACS. FACS analysis revealed that the percentage of GFP-positive cells that were AP-positive significantly decreased when compared with GFP-negative cells following exposure to 1,3 and 6 mM of H_2_O_2_ (GFP- positive % vs. GFP- negative % at 0 mM = 3.2 vs. 2.1; at 1 mM = 3.6 vs.9.7; at 3 mM = 13.3 vs. 38.3; at 6 mM = 13.6 vs.49.9; at 9 mM = 28.1 vs. 66.3) (n = 5, P<0.05) (Fig. 6). These findings indicated that GRP78 overexpressing cells could survive longer than non-GRP78 overexpressing cells following H_2_O_2_ exposure.

### Evaluation of GRP78 Overexpression on Cell Lipid Peroxidation Following H_2_O_2_ Exposure

**Figure 7:**
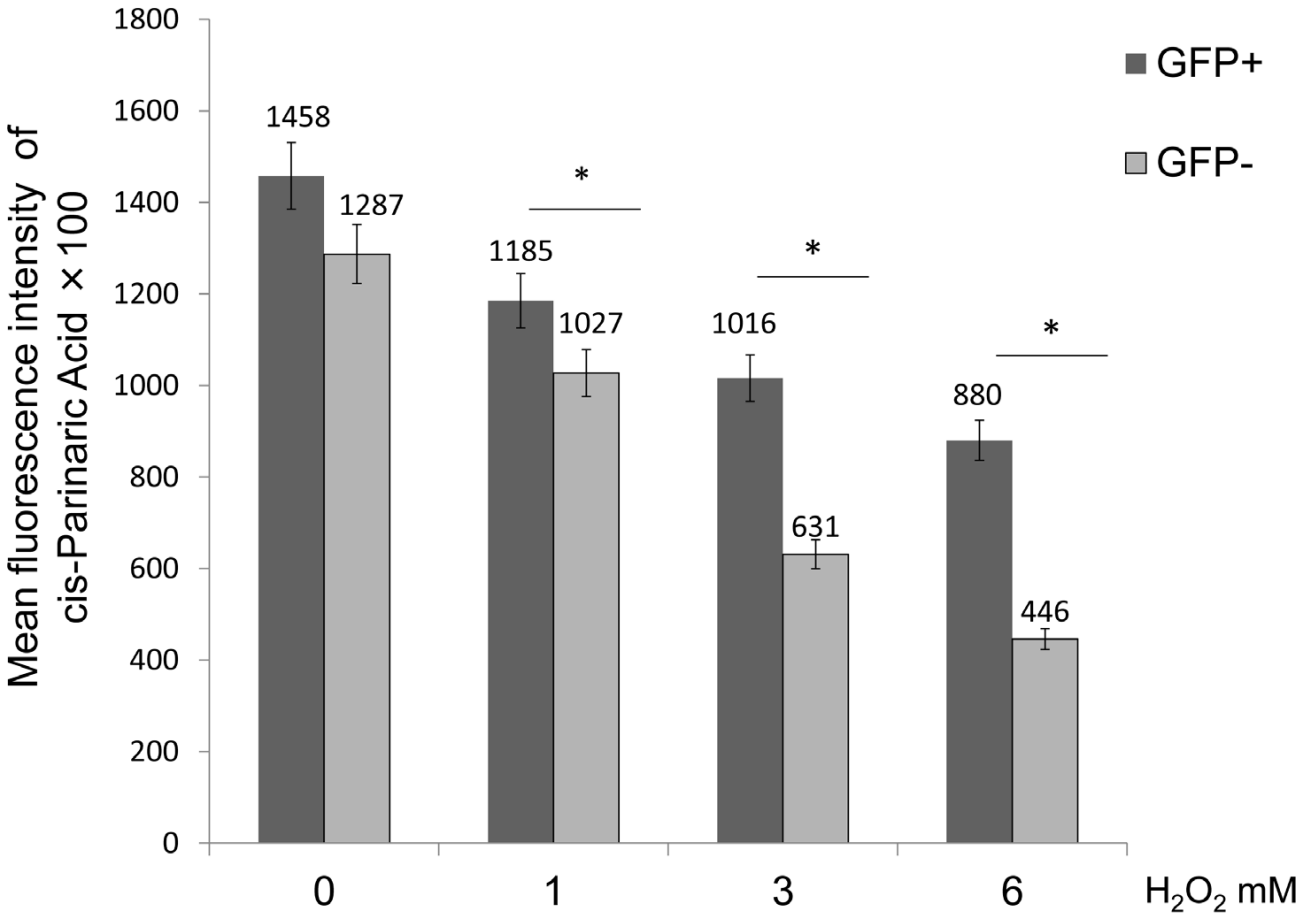
Evaluation of GRP78 overexpression on cell lipid peroxidation following H_2_O_2_ exposure. FACS analysis of the mean fluorescence intensity of cis-Prinaric acid in GFP-negative and -positive cells following treatment with H_2_O_2_. The cis-Parinaric Acid mean fluorescence intensity of GFP-positive cells was significantly higher when compared with GFP-negative cells following treatment with 1, 3 and 6 mM H_2_O_2_ (*P<0.05).

As described above, a mixture of cells consisting of GFP-positive and -negative cells were treated with H_2_O_2_ 1–6 mM for 6 h, and labeled with cis-Pariaric acid to evaluate lipid peroxidation of cell membranes. FACS revealed that the mean fluorescence intensity of cis-Pariaric acid in GFP-positive cells was significantly higher than that in GFP-negative cells following H_2_O_2_ exposure at 1, 3 and 6 mM (GFP-positive vs. GFP-negative at 0 mM = 1458 vs. 1287; at 1 mM = 1185 vs.1027; at 3 mM = 1016 vs. 631; at 6 mM = 880 vs. 446) (n = 5, P<0.05) (Fig. 7). These findings indicate that GRP 78 overexpressing cells can suppress lipid peroxidation more than non-GRP78 overexpressing cells following H_2_O_2_ exposure.

### GSH Expression Levels in GRP78 Overexpressing Cells Following H_2_O_2_ Treatment

**Figure 8:**
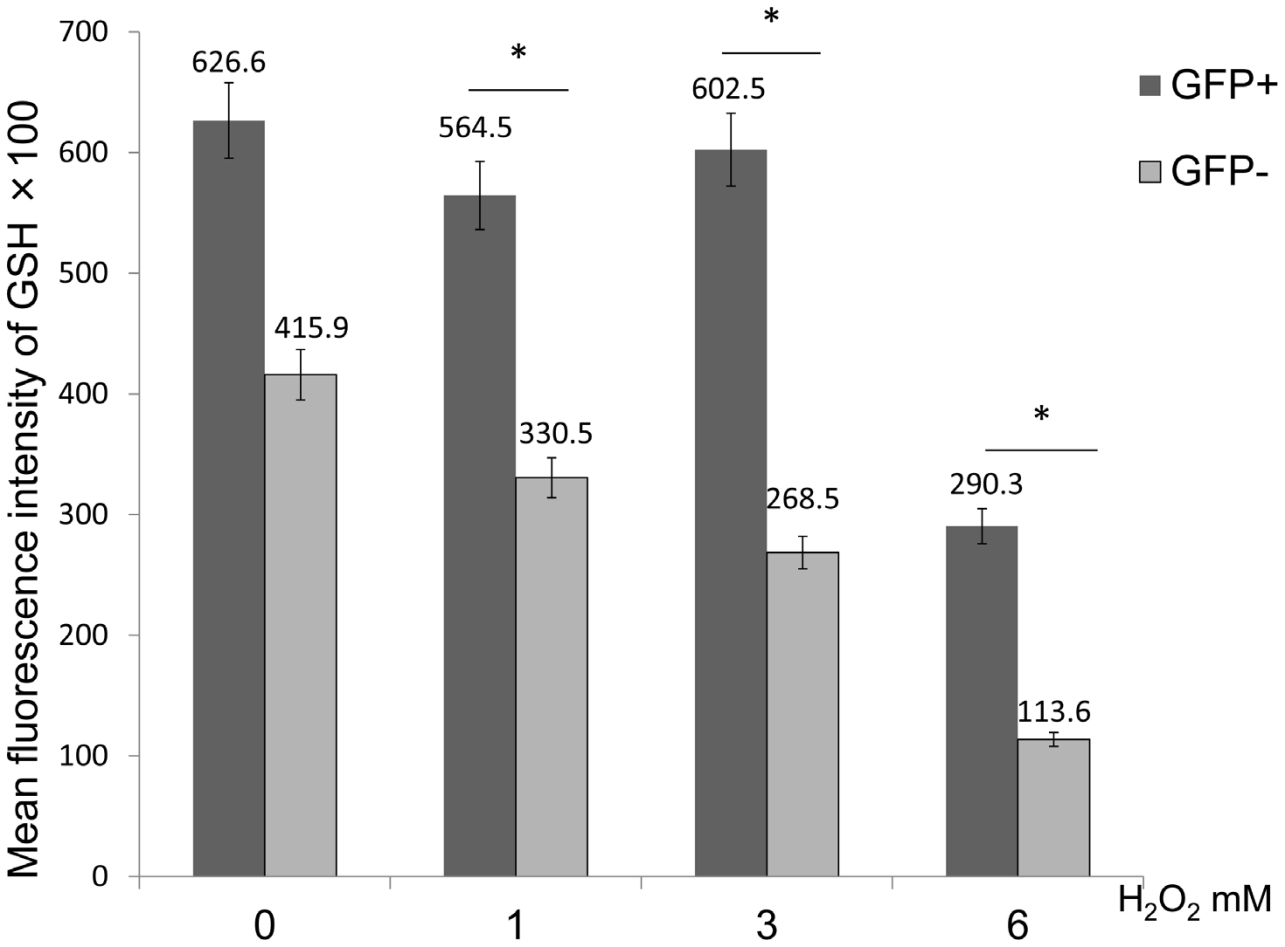
GSH expression levels in GRP78 overexpressing cells following H_2_O_2_ treatment. FACS analysis of the mean fluorescence intensity of GSH in GFP-negative and -positive cells following treatment with H_2_O_2_. The GSH mean fluorescence intensity of GFP-positive cells was significantly higher when compared to GFP-negative cells following treatment with 1, 3 and 6 mM H_2_O_2_ (*P<0.05).

We observed GSH expression levels in glutathione using FASC analysis. FACS revealed that the mean fluorescence intensity of GSH in GFP-negative cells significantly decreased when compared with GFP-positive cells following exposure to 1, 3 and 6 mM of H_2_O_2_ (GFP-positive vs. GFP-negative at 0 mM = 62660 vs. 41590; at 1 mM = 56450 vs. 33050; at 3 mM = 60250 vs. 26850; at 6 mM = 29030 vs. 11360) (n = 5; P<0.05) (Fig. 8). Thus, intracellular GSH expression levels in GRP78 overexpressing cells were significantly higher than that of non-GRP78 overexpressing cells.

### Evaluation of NQO1 Expression Levels in GRP78 Overexpressing Cells Following H_2_O_2_ Exposure

**Figure 9:**
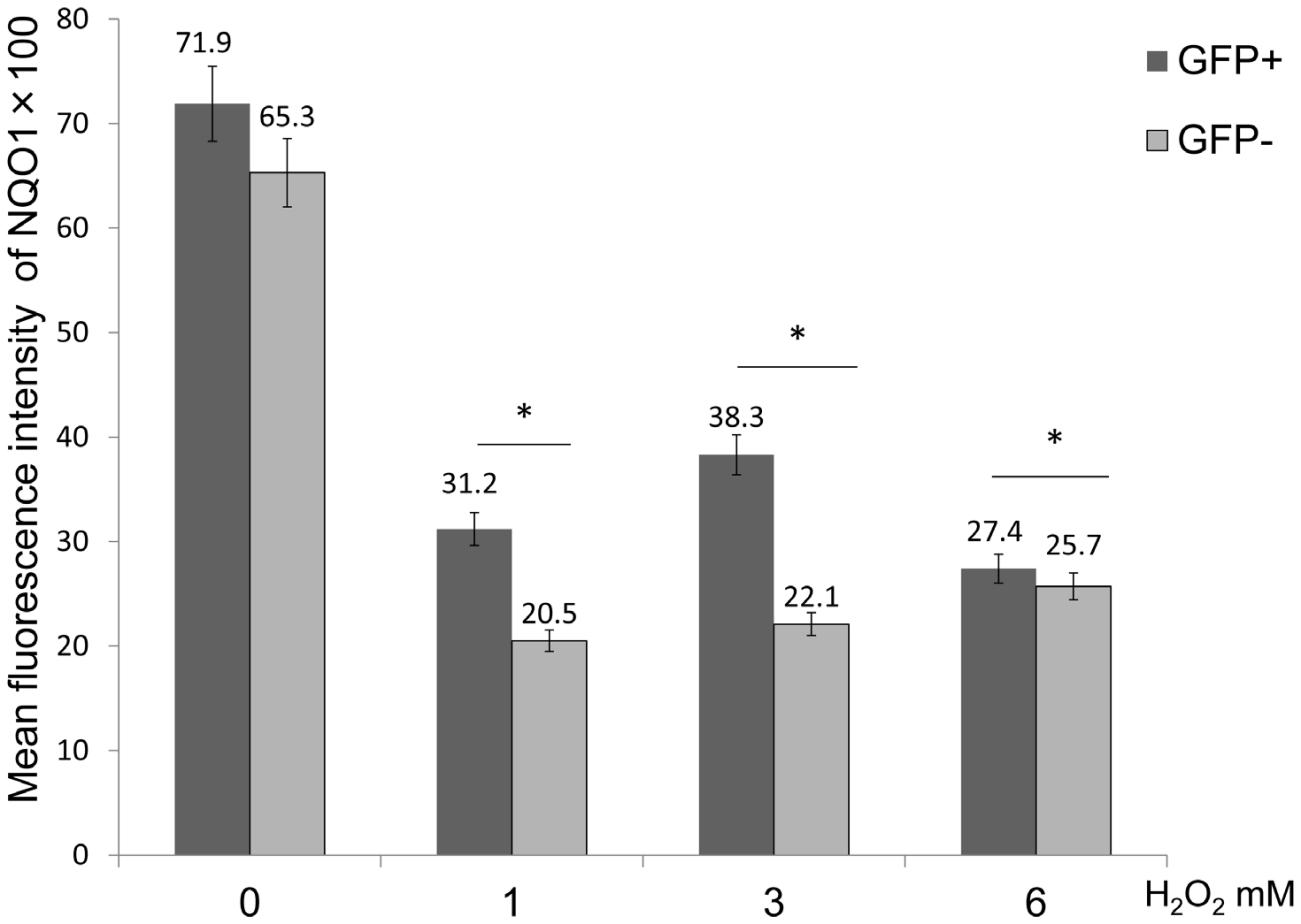
Evaluation of NQO1 expression levels in GRP78 overexpressing cells following H_2_O_2_ exposure. FACS analysis of the mean fluorescence intensity of NQO1 in GFP-negative and -positive cells following treatment with H_2_O_2_. The NQO1 mean fluorescence intensity of GFP-positive cells was significantly higher when compared to GFP-negative cells following treatment with 1, 3 and 6 mM H_2_O_2_ (*P<0.05).

The mean fluorescence intensity of NQO1 in GFP-negative cells significantly decreased when compared with GFP-positive cells following 1, 3 and 6 mM of H_2_O_2_ (GFP- positive vs. GFP-negative at 0 mM = 7190 vs.6530; at 1 mM = 3120 vs. 2050 50; at 3 mM = 3830 vs. 2210; at 6 mM = 2740 vs. 2570) (n = 3, P<0.05) (Fig. 9). These result showed that levels of NQO1 in GRP78 overexpressing cells were higher than non-GRP78 overexpressing cells.

## Discussion

GRP78 levels have been reported to increase in cells following cytotoxic-induced ER stress, where it contributes to cell survival [11], [12], [28]. According to reports that metals are implicated in the etiology or pathogenesis of Alzheimer’s disease, some metals such as lead (Pb) induce the expression of GRP78, which is often associated with oxidative stress, and Pb impairs GRP78 function following binding [29]. Moreover, it has been reported that GRP78 may play a role in the modulation of the sensitivity of cells to stress after oxidative injury. Increases in the mRNA expression of GRP78 are observed in retinal pigment epithelial cells exposed to oxidative stress [17]. Experiments using cultured neurons reveal that GRP78 may protect cells against oxidative stress via actions involving mainly the maintenance of calcium homeostasis [16]. Meanwhile, it was reported that GRP78 expression did not increase when cells were exposed to H_2_O_2_, which suggested that H_2_O_2_ exposure may not induce the ER stress pathway [24]. In our study, an increase in GRP78 expression in C6 cells was not observed after treatment with H_2_O_2_, however, the viability of cells decreased. Considering previous reports, our results suggest that GRP78 itself could play an important role in protecting cells against H_2_O_2_ injury regardless of whether the pathways that mediate GRP78 expression respond to their extracellular stimuli.

H_2_O_2_ causes cytotoxicity via the formation of more potent oxidants including OH·, which causes lipid peroxidation of the cell membrane [7], [30]. Lipid peroxidation disrupts the normal structure of cellular and subcellular membranes. In addition, the process produces byproducts such as 4-hydroxynonenal (4-HNE) or acrolein, both of which bind to proteins and damage their structure and function [30], [31]. The present results show that GRP78 overexpressing cells suppress lipid peroxidation and may contribute to cell survival following H_2_O_2_ treatment. These data suggest that GRP78 can promote the expression of some antioxidants and may contribute to the protection of cells against H_2_O_2_ injury.

While a number of antioxidants are involved in the detoxification of H_2_O_2_, GSH is the primary defense against H_2_O_2_ [18]. GSH inhibits lipid peroxidation initiation by scavenging OH· or other ROS. Moreover, GSH also serves as a co-factor for GSH peroxidases that remove H_2_O_2_ [20], [22]. It was reported that GSH was useful for curtailment of lipid peroxidation damage in acute spinal cord injury [20]. The ratio of GSH reduces when an increase in ROS induces ER stress [31], [32]. In our results, when cells were exposed to H_2_O_2_, GSH expression in GRP78 overexpressing cells was high when compared with non-GRP78 overexpressing cells. These results suggest that the increase of GRP78 by gene transfection may contribute to the increase in GSH or inhibit GSH consumption, thus leading to cell survival.

We observed the influence of GRP78 on NQO1 in this study. NQO1 catalyzes the electron reduction of quinone and quinoid compounds to hydroquinones, thus limiting the formation of semiquinone radicals, and the subsequent generation of ROS [19], [33]. According to reports regarding the role of NQO1, H_2_O_2_-dependent formation of reactive oxygen intermediates was shown to be reduced following treatment with neuroprotective agents that induce NQO1 expression [34], [35]. In our results, NQO1 expression levels in GRP78 overexpressing cells was higher than in non-GRP78 overexpressing cells. This phenomenon continued following H_2_O_2_ exposure. These findings indicate that GRP78 may be advantageous to the expression of NQO1.

NQO1 has the ability to use both NADPH and NADH equally efficiently, thus NQO1 may contribute to the regulation of the redox balance by modulating reduced/oxidized pyridine nucleotide ratios [36]. NADPH is required to reduce oxidized glutathione (GSSG) to GSH [37]. This NQO1 role appears not to contradict our results that GSH expression levels in GRP78 overexpressing cells were higher than non- GRP78 overexpressing cells.

Taken together, this present study suggests that GRP78 plays an important role in protecting glial cells against H_2_O_2_ toxicity by regulating GSH and NQO1 expression. However, there are several pathways and factors related to GRP78 expression in cells and further studies are required to understand the mechanisms involved and the direct relationship between GRP78, GSH and NQO1 in order for molecular/pharmacological treatments of neurotrauma or neurodegenerative diseases to be developed.
